# Effects of Single-Joint Type Hybrid Assistive Limb in Knee Rehabilitation on Reduction of Pre-motor Time and Increased Rate of Fast Muscle Activation for Anterior Cruciate Regiment (ACL) Reconstruction

**DOI:** 10.7759/cureus.68967

**Published:** 2024-09-08

**Authors:** Yuichiro Soma, Hirotaka Mutsuzaki, Tomokazu Yoshioka, Shigeki Kubota, Koichi Iwai, Yukiyo Shimizu, Akihiro Kanamori, Masashi Yamazaki

**Affiliations:** 1 Department of Orthopedic Surgery, Institute of Medicine, University of Tsukuba, Tsukuba, JPN; 2 Department of Physical Medicine and Rehabilitation, Institute of Medicine, University of Tsukuba, Tsukuba, JPN; 3 Department of Orthopedic Surgery, Ibaraki Prefectural University of Health Sciences, Ami, JPN; 4 Department of Physical Medicine and Rehabilitation, Ibaraki Prefectural University of Health Sciences, Ami, JPN; 5 Department of Rehabilitation Medicine, University of Tsukuba Hospital, Tsukuba, JPN

**Keywords:** acl reconstruction, knee hal single-joint training, peak amplitude time, pre-motor time, rate of emg rise, robot assistive training

## Abstract

Background/objectives: Changes in muscle characteristics after post-anterior cruciate regiment (ACL) reconstruction are common. Knee robotic-assisted therapy using hybrid assistive limb (HAL) single-joint training for recovery from ACL injury has the potential to optimize muscle activity; however, its neurophysiological effects remain unclear. Thus, this study aimed to explore the electrophysiological parameters.

Methods: This prospective, nonrandomized, controlled trial was conducted between December 2021 and January 2024. The patients were divided into two groups: the HAL group and the control group, each including five patients who underwent arthroscopic ACL reconstruction. Knee HAL single-joint training was conducted once weekly for three sessions. Three electrophysiological measures were examined related to knee neuromuscular responses pre-motor time, peak amplitude time, and neuromuscular rate of electromyography rise (RER) using surface electromyography (EMG). The pre-motor time and peak amplitude time were assessed in both groups at each session pre- and post-intervention. Both groups were evaluated for RER at postoperative weeks 17 and 21.

Results: Regarding the interaction of pre-motor time within each group, the interaction of pre-motor time within each group, the effect size of vastus medialis (VM) was larger in the HAL group. The peak amplitude time of EMG, the overall estimated marginal means, and the HAL group exhibited a significant difference in the VM (p=0.019), while vastus lateralis (VL) showed no significant difference but a larger effect size (d=0.61). The RER revealed a significant difference in semitendinosus-RER30ms in the HAL group (p=0.044).

Conclusions: The knee HAL training for post-ACL reconstruction patients may influence neurophysiological outcomes.

## Introduction

Anterior cruciate regiment (ACL) injuries can disrupt the intricate interactions within the neuromuscular system, resulting in compromised kinesthesia and proprioception, abnormal muscle activation, and diminished stability of the dynamic knee joint [1]. ACL reconstruction often leads to alterations in the physiological characteristics of muscles. A previous study illustrated that the prolonged total reaction time to a visual stimulus in the atrophied quadriceps femoris muscle of humans after ACL reconstruction signifies an extension of the electromechanical delay [2]. The extended reaction time resulting from muscle atrophy may be attributed to peripheral physiological neuromuscular irregularities that are not limited solely to impaired proprioception [2]. ACL-related reductions in muscle strength are believed to stem from both quantitative decreases in muscle mass and muscle efficiency [3]. This loss of muscle efficiency has been associated with factors such as neuromuscular function and muscle stiffness [4].

Based on biopsy findings, the extent of these alterations is more pronounced in muscles characterized by a higher proportion of type II fibers [5]. Slower muscle contractile properties compromise excitation-contraction coupling [6] and modifications to muscle architecture [7], as well as changes in musculotendinous stiffness [8], and may exert varying influences on rapid muscle contractions as opposed to slow or static muscle contractions. Neuromuscular activation refers to the operational process through which the nervous system generates muscular force, encompassing the recruitment and rate-coding of motor units [9]. Surface electromyography (EMG), which detects the bioelectrical activity associated with muscle contractions, is an extensively utilized method for assessing neuromuscular activation [10]. In a previous study, older individuals exhibited lower relative rapid force characteristics than their younger counterparts. Interestingly, no substantial differences were observed in the rates of muscle activation, suggesting the influence of qualitative factors. These factors include type II fiber atrophy, increased accumulation of intramuscular fat and connective tissue, a reduced pennation angle, and altered fascicle length [11]. Notably, there have been relatively few investigations on the impact of muscle activation rates following ACL reconstruction.

A previous study has suggested that robot-assisted therapy rehabilitation with a proposed algorithm detects the motion intention in the knee joint and requires no prior training with EMG signals [12]. However, there are limited reports of papers on the application of robot-assisted therapy rehabilitation post-ACL reconstruction. The hybrid assistive limb (HAL) offers the wearer a feedback system that enables them to monitor their real-time bioelectrical signals on display during HAL training. In neurorehabilitation, voluntary and active training using HAL is essential for errorless learning and motor-skill acquisition [13,14]. Recent studies have reported alterations in neural activity attributed to HAL [15,16]. It is postulated that HAL assistance, relying on bioelectric signals, may have contributed to the induction of non-invasive modulation of the central nervous system's control of muscles [17]. Previous studies focusing on HAL single-joint training indicated that sensory feedback, resulting from a motion that aligns with physiological expectations, has the potential to induce modifications in the central nervous system [18]. In a previous investigation involving knee HAL single-joint training for patients after ACL reconstruction, substantial differences in muscle strength were observed compared with the control group across various velocities in isokinetic muscle strength tests [19]. Despite the available evidence suggesting that knee HAL single-joint training can positively impact muscle strength recovery, enhance muscle activity efficiency, and lead to improved outcomes in physical evaluations, its effects on neurophysiological aspects remain unclear. Our hypothesis posited that knee HAL single-joint training would result in subtle alterations in pre-motor time and an increased rate of rapid muscle activation. We aimed to explore electrophysiological parameters, including pre-motor time, peak amplitude time, and neuromuscular activation rate on EMG compared with the control group.

## Materials and methods

Exploratory study

We performed a prospective trial at the University of Tsukuba Hospital, Tsukuba, Japan, between December 2021 and January 2024. This exploratory study first started with the HAL group, followed by the control group. During this period, all patients who underwent arthroscopic ACL reconstruction were assessed for eligibility. The inclusion criteria for the HAL group were as follows: a primary ACL injury, the ability to understand an explanation of the study and provide informed consent, and the availability of observations throughout the study. Patients with multiple knee ligament injuries, those for whom wearing and training using the HAL was expected to be difficult because of the underlying disease, and those with perioperative complications were excluded. The inclusion criteria of the control group were a primary ACL injury; the ability to comprehend the study’s explanation and provide informed consent; and consistent availability for the study’s duration. Patients with multiple knee ligament injuries and those who experienced perioperative complications were excluded. The semitendinosus (ST) tendon alone or both the ST and gracilis tendons were harvested and used as multistranded grafts. The tendon graft was attached to an ultra-button adjustable-loop device (Smith & Nephew Endoscopy, Andover, USA) on the femoral side. The tibial end of the graft was sutured using a double-spike plate and screw (Smith & Nephew Endoscopy) with an initial tension of 20 N or 30 N measured using a tension meter at 20° or 30° knee flexion. Femoral and tibial bone tunnels were created anatomically at the tibial and femoral insertions of the ACL using the outside-in tunnel technique.

**Figure 1:**
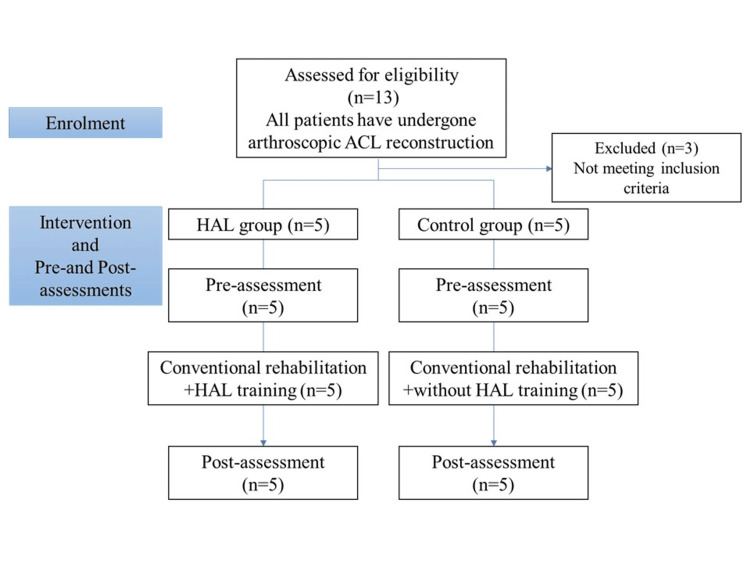
Flow of participants through the study protocol HAL: hybrid assistive limb; ACL: anterior cruciate regiment

All patients underwent rehabilitation training by a physical therapist after surgery starting on postoperative day 1. Range of motion (ROM) was fixed between 0° and 90° and weight-bearing exercises were initiated on postoperative day 5. After discharge, the rehabilitation program was administered once weekly by either a physical therapist or an athletic trainer. The program included closed and open kinetic chain exercises, strength training of the hip and knee muscles, neuromuscular training, neuromuscular electrical stimulation, and cryotherapy for the ACL-reconstructed leg. After a three-month follow-up, patients engaging in competitive sports continued with the rehabilitation program twice or thrice monthly, while those participating in recreational sports received it once monthly. The frequency and content of inpatient and outpatient rehabilitation following ACL were similar in both groups. We chose not to control the rehabilitation program to maintain the external validity of our findings. Figure 1 summarizes the flow of participants through the study protocols.

The study was approved by the Ethics Committees of Tsukuba University Faculty of Medicine (approval number: TCRB18-077) and Ibaraki Prefectural University of Health Sciences (approval code: e119) and was performed in accordance with the Declaration of Helsinki. This study was registered with the Japan Registry of Clinical Trials (jRCT) Clinical Trials Registry (CRB3180028).

All patients provided written informed consent before enrollment in the study.

Knee HAL single-joint training

Knee HAL single-joint training commenced 18 weeks after ACL reconstruction to mitigate the risk of graft tension laxity, partial graft tear, and poor synovial coverage [20,21]. Knee HAL single-joint training was conducted once weekly for three sessions. Before setup, the physical therapist measured the femoral and lower limb lengths, hip and ankle widths, and maximum active flexion and extension ROM of the ACL-reconstructed leg. While the patient was seated, the therapist fitted each leg attachment and ankle support (fitting time: 3-5 minutes). After measuring the maximum active flexion and extension angles, the knee HAL assist angle was set to prevent overassistance before the intervention. Regarding extension, the HAL assist flexion angle was adjusted to -5° from the maximum active extension angles. Conversely, the HAL assist flexion angle was set to 120°, corresponding to the maximum flexion angle. All the patients were adjusted to this angle. Surface electrodes were attached to the quadriceps and hamstring muscles to evaluate bioelectrical activity from the long axis (along the belly) of each muscle. We checked whether muscle contraction occurred in the waveform of the electromyogram while extending and flexing the knee joint with the HAL. To set the knee HAL single-joint training assistance level before the first knee HAL single-joint training session, we instructed the patients to perform leg extension and flexion movements comfortably and smoothly, following their voluntary movement patterns. Subsequently, we determined the knee HAL single-joint training assistance level [22]. During the knee extension training, the patient was seated at the end of the bed. For knee flexion training, the patient remained in the prone position on the bed. Five sets of knee HAL single-joint training-assisted knee extension and flexion exercises were performed (10 exercises per set for a total of 50 exercises) [19,22]. Each session lasted approximately 50 minutes, including the fitting and evaluation. Conventional rehabilitation was performed on days without HAL training.

Electrophysiological assessments

**Figure 2:**
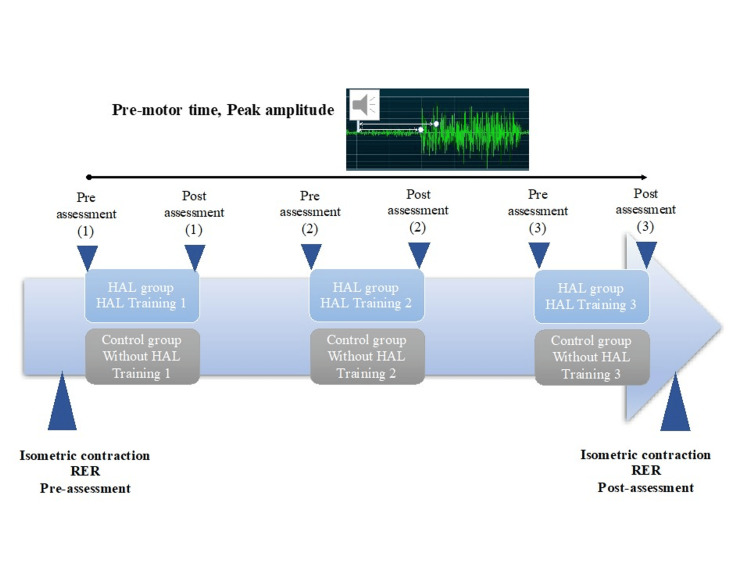
Experimental protocol for both the HAL and control groups for pre-motor time, peak amplitude time, and RER assessments RER: rate of electromyography rise; HAL: hybrid assistive limb

**Figure 3:**
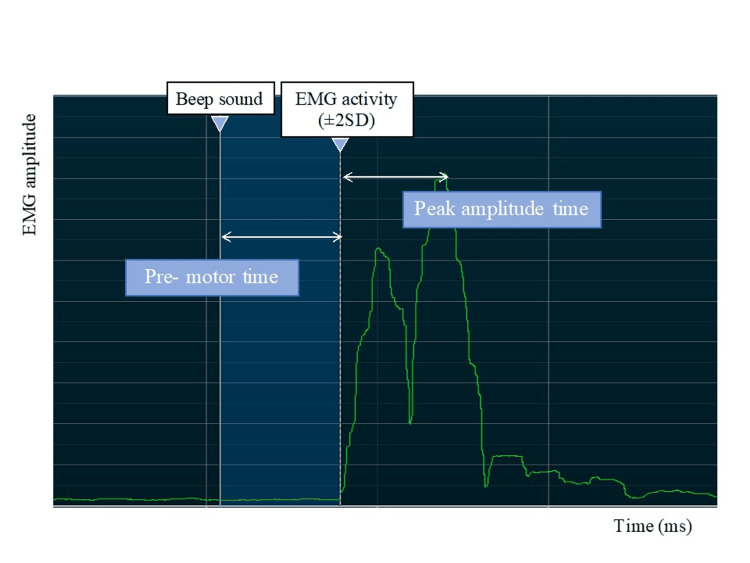
An example of a pre-motor time and peak amplitude time on EMG EMG: electromyography

**Figure 4:**
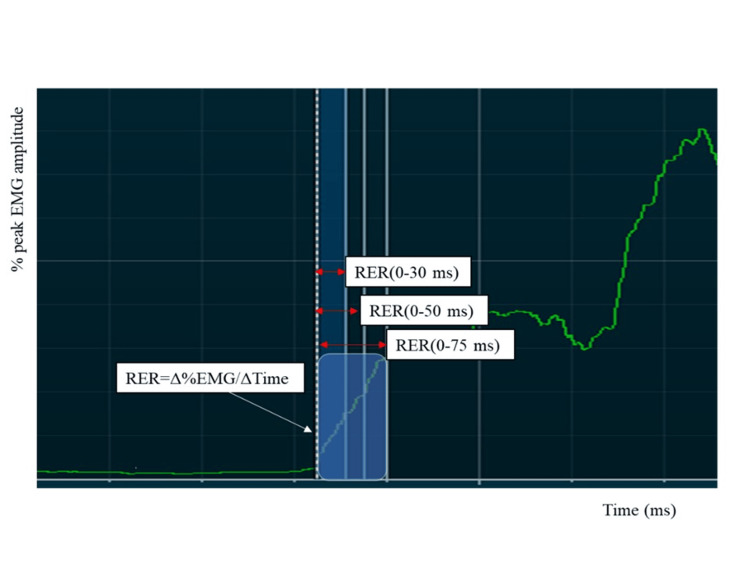
An example of a RER at 0-30, 0-50, 0-75 ms from onset RER: rate of electromyography rise; EMG: electromyography

This study investigated three electrophysiological measurements (pre-motor time, peak amplitude time, and neuromuscular activation rate) related to the neuromuscular response of the knee during EMG rise. Both the HAL and control groups were assessed for rate of electromyography rise (RER) at postoperative weeks 17 and 21. The pre-motor time and peak amplitude time in both groups were assessed at each session immediately before and after the intervention. In the control group, participants performed knee extension and flexion exercises without HAL assistance. The exercise regimen involved 10 exercises per set for a total of 50 exercises. The control group was utilized to ensure that the assessments could be conducted under comparable conditions, enabling a meaningful comparison between the effects of knee HAL training and traditional exercises in patients after ACL reconstruction (Figure 2). The measuring system used was a four-channel MyoSystem EMG unit (Noraxon, Scottsdale, USA) with bipolar Ag-AgCl surface electrodes, each measuring 1 cm in diameter and having a center-to-center distance of 2.5 cm. The speed and amplitude of the anteroposterior neuromuscular response of the knee are crucial for ACL protection [23]. Pre-motor time, defined as the time to onset of muscle activity, was measured as the EMG amplitude exceeding the resting mean plus 2SD [24] in the vastus medialis (VM), vastus lateralis (VL), biceps femoris (BF), and ST. Electrode placement on the examined muscles followed the EMG for non-invasive assessment of muscle recommendations [25]. Peak amplitude time was measured in the VM, VL, BF, and ST and defined as the time from each muscle's pre-motor time to its peak amplitude in the normalized EMG signals (Figure 3). The participants were instructed to perform leg extension and flexion movements quickly, following an auditory cue. Leg extension movements were performed while the patient was seated, whereas leg flexion movements were performed with the patient in a prone position on the bed. The rate of EMG rise was calculated for each muscle as the mean derivative of the normalized EMG signal between pre-motor time and movement onset, represented as the linear slope of the normalized EMG-time curve (RER=ΔEMG/Δtime) at time intervals of 30 ms (RER30), 50 ms (RER50), and 75 ms (RER75) (Figure 4) [26,27]. To measure the rate of the EMG increase, the participants used a dynamometer (Biodex System III; Biodex Medical Systems, Sakai Inc., Tokyo, Japan). They were seated on a dynamometer chair, secured with straps at the thigh, pelvis, and torso, with the hip joint flexed to 80°and the knee joint flexed to 60°. During the testing phase, the participants were familiarized with the procedures. Each participant made maximal attempts at knee extension and flexion. A three-minute rest was provided between each attempt. The test involved participants exerting isometric maximum voluntary leg extension and flexion contractions for three seconds. They were instructed to exert force '‘as strongly and as quickly as possible.’' Online visual feedback and strong verbal encouragement were provided during testing to facilitate maximal effort [28].

Data analysis

MyoResearch 3 software was utilized for EMG signal processing analysis. A sampling rate of 1500 Hz and a bandpass filter (10-500 Hz) were used. EMG signals were rectified using a root mean square filter of 100 ms to evaluate EMG amplitudes. The amplitude of each muscle was normalized to the percentage of its isometric maximal voluntary contraction (MVC). All contractions were performed while seated in the Biodex System III according to the MVC protocol [26,27].

Statistical analysis

Initially, to determine the differences in the characteristics of the two groups, a t-test was conducted for numerical data, and Fisher's exact probability test was employed for categorical data. Subsequently, for the EMG data, the differences in the means between the pre- and post-conditions were assessed within each group. The Shapiro-Wilk test was performed to assess the normality of the data. When a normal distribution was assumed, a corresponding t-test was employed; otherwise, the Wilcoxon signed-rank test was used. Effect size (Cohen's d) was also calculated [29]. Furthermore, a Generalized Linear Mixed Model (GLMM) was employed to examine attributes of participants, including age, sex, body mass index (BMI), athletic level, and side of injury in relation to each set of EMG data. In evaluating the model’s goodness of fit, lower values for corrected Akaike's Information Criterion Corrected (AICc) and Schwarz's Bayesian Information Criterion (BIC) indicate a better fit. The results, ordered from left to right, demonstrate the models with a superior fit. Additionally, the effects of the group (HAL group/control group), measurement period (pre/post), the interaction between the group and measurement period (pre-post group), and the number of measurements were examined. The analysis was conducted using the overall estimated marginal means between pre- and post-intervention for each group based on the estimated mean. Analyses were conducted using IBM SPSS Statistics for Windows, Version 29 (Released 2021; IBM Corp., Armonk, New York, USA) with a significance level of 5% for all tests.

## Results

The HAL group included five patients (three male and two female patients; mean age: 20.4±4.0 years; height: 168.3±12.1 cm; weight: 66.5±19.4 kg) who underwent arthroscopic ACL reconstruction with soft-tissue graft materials (anatomic single-bundle). The control group included five patients (one male and four female patients; mean age: 21.0±1.2 years; height: 158.4±12.3 cm; weight: 61.4±10.6 kg) who had undergone arthroscopic ACL reconstruction with soft-tissue graft materials (anatomic single-bundle). Table 1 summarizes the patients’ clinical characteristics.

**Table 1:** Patient clinical characteristics Numerical data were analyzed using t-tests, whereas categorical data were analyzed using Fisher's exact probability tests. HAL: hybrid assistive limb; BMI: body mass index

	HAL group	Control group	p-value
Age (year)	20.4±4.0	21.0±1.2	0.755
Sex (male/female)	3/2	1/4	0.524
Height (cm)	168.3±12.1	158.4±12.3	0.235
Weight (kg)	66.5±19.4	61.4±10.6	0.625
BMI (kg/m^2^)	23.2±5.6	24.6±4.5	0.678
Injured side (right/left)	1/4	2/3	1.000
Graft materials (single-bundle/double-bundle)	5/0	5/0	-
Sports level (competitive/recreational)	2/3	3/2	1.000

Table 2 presents the pre-motor time on EMG for both the HAL and control groups. Neither group exhibited statistical significance in the pre- and post-intervention assessments. Nevertheless, it is notable that only the HAL group demonstrated moderate and large effect sizes for VM, BF, and ST.

**Table 2:** Pre-motor time on electromyography for the HAL and control groups The means and corresponding standard deviations are presented. Paired t-tests were employed for the conducted tests, while Wilcoxon's signed-rank test was utilized when applicable (^*^). Effect sizes were computed using Cohen's d. HAL: hybrid assistive limb; post: post-assessment; pre: pre-assessment; VM: vastus medialis; VL: vastus lateralis; BF: biceps femoris; ST: semitendinosus

		HAL group		p-value	Effect size (d)	Control group		p-value	Effect size (d)
		Pre	Post			Pre	Post		
VM	Session 1	0.426±0.05	0.406±0.05	0.517	0.317	0.438±0.08	0.388±0.07	0.444	0.134
	Session 2	0.460±0.08	0.412±0.05	0.103	1.162	0.444±0.10	0.384±0.05	0.232	0.095
	Session 3	0.490±0.15	0.426±0.08	0.228	0.635	0.357±0.09	0.475±0.08	0.138^*^	0.149
VL	Session 1	0.414±0.06	0.395±0.06	0.567	0.278	0.436±0.08	0.393±0.06	0.489	0.126
	Session 2	0.418±0.062	0.448±0.06	0.453	0.371	0.444±0.10	0.383±0.04	0.257	0.102
	Session 3	0.445±0.06	0.403±0.06	0.279	0.559	0.352±0.07	0.468±0.08	0.142	0.142
ST	Session 1	0.456±0.07	0.343±0.05	0.113	1.108	0.416±0.06	0.379±0.04	0.293	0.068
	Session 2	0.383±0.07	0.360±0.07	0.281	0.557	0.400±0.07	0.376±0.12	0.611	0.097
	Session 3	0.419±0.06	0.370±0.04	0.190	0.706	0.405±0.08	0.317±0.08	0.113	0.097
BF	Session 1	0.457±0.07	0.346±0.06	0.154	0.947	0.437±0.06	0.383±0.04	0.171	0.073
	Session 2	0.398±0.07	0.371±0.07	0.180	0.726	0.410±0.06	0.3827±0.11	0.577	0.106
	Session 3	0.426±0.06	0.375±0.03	0.175	0.737	0.413±0.09	0.332±0.07	0.151	0.101

In Table 3, model 1 was formulated for EMG pre-motor time, incorporating age, gender, BMI, sports level, injured side, group, pre-post, interaction between pre-post and group, and session as fixed effects and the patient-id as the random effect. GLMM analysis yielded significant findings for age and BMI in the case of VM. Subsequently, model 2 was constructed, excluding variables that did not reach significance, incorporating fixed effects for age, BMI, and interactions between group, pre-post, and pre-post and group. A similar analysis was carried out for VL, where no significant variables were identified in model 1, but age and BMI remained significant in model 2. For BF, only pre-post exhibited significance in model 1, and the results of model 2 were consistent. Notably, across the four pre-motor times, the values for AICc and BIC were smaller in model 2; given that smaller values are preferable, the results from model 2 were adopted in all instances. These outcomes suggest that individual characteristics like age and BMI hold significance for VM and VL, while pre-post changes are noteworthy for ST and BF. Notably, no significant differences between groups were observed in this study.

**Table 3:** GLMM of pre-motor time on EMG for the HAL and control groups ^*^: significant difference (p<0.05) AICc: Akaike's Information Criterion Corrected; BIC: Schwarz's Bayesian Information Criterion; group: effects of group (HAL group/control group); pre-post: measurement period; pre-post group: interaction between group and measurement period; VM: vastus medialis; VL: vastus lateralis; BF: biceps femoris; ST: semitendinosus; BMI: body mass index; GLMM: Generalized Linear Mixed Model; HAL: hybrid assistive limb

Variables	VM				VL				ST				BF			
	Model 1		Model 2		Model 1		Model 2		Model 1		Model 2		Model 1		Model 2	
	t-value	p-value	t-value	p-value	t-value	p-value	t-value	p-value	t-value	p-value	t-value	p-value	t-value	p-value	t-value	p-value
Intercept	2.841	0.007	4.254	0.001	3.338	0.002	4.704	0.001	2.099	0.123	3.573	0.011	2.616	0.073	4.506	0.004
Age	2.997	0.004^*^	2.658	0.010^*^	1.566	0.124	2.335	0.023^*^	0.851	0.456	0.959	0.374	1.185	0.319	1.292	0.244
Sex	-0.050	0.960	-	-	0.317	0.753	-	-	-0.256	0.814	-	-	-0.292	0.788	-	-
BMI	-3.815	0.001	-3.240	0.002^*^	-1.881	0.066	-2.419	0.019^*^	-1.026	0.380	-1.122	0.305	-1.386	0.260	-1.634	0.153
Sports level	0.144	0.886	-	-	-0.637	0.527	-	-	0.213	0.845	-	-	0.620	0.576	-	-
Injured side	-1.455	0.152	-	-	0.924	0.360	-	-	-0.571	0.607	-	-	-0.846	0.458	-	-
Group	1.349	0.184	1.385	0.172	0.146	0.885	0.505	0.616	0.216	0.840	0.198	0.847	0.254	0.811	-0.011	0.992
Pre-post	0.074	0.941	0.074	0.942	0.159	0.874	0.164	0.870	-2.227	0.031^*^	-2.248	0.029^*^	-2.348	0.023^*^	-2.377	0.022^*^
Pre-post group	-0.879	0.384	-0.874	0.386	-0.390	0.698	-0.403	0.688	-0.178	0.860	-0.208	0.836	-0.037	0.970	-0.072	0.943
Session 3	0.904	0.370	-	-	0.336	0.738	-	-	-0.997	0.324	-	-	-0.905	0.371	-	-
Session 2	0.767	0.447	-	-	0.606	0.547	-	-	-0.888	0.379	-	-	-0.730	0.469	-	-
AICc	-69.324		-90.953		-82.827		-109.832		-89.296		-113.474		-87.534		-112.587	
BIC	-65.849		-87.253		-79.305		-106.090		-85.821		-109.773		-84.058		-108.887	

Concerning the interaction, we assessed the contrast between pre and post in each group using the estimated mean (Table 4). Additionally, effect sizes were computed using Cohen's d. The overall estimated marginal means showed significant improvement from pre-treatment to post-treatment. Significant differences were observed in ST and BF in both groups, with no significant differences in VM. Notably, the effect size was larger in the HAL group (d=0.72) for VM despite the absence of statistical significance.

**Table 4:** The overall pre-motor time on EMG estimated marginal means from pretreatment to posttreatment for the HAL and control groups ^*^: significant difference (p<0.05) HAL: hybrid assistive limb; post: post-assessment; pre: pre-assessment; VM: vastus medialis; VL: vastus lateralis; BF: biceps femoris; ST: semitendinosus; d: effect sizes were computed using Cohen's d

	HAL group		p-value	Effect size (d)	Control group		p-value	Effect size (d)
	Pre	Post			Pre	Post		
VM	0.457	0.423	0.254	0.72	0.416	0.418	0.942	0.04
VL	0.424	0.414	0686	0.25	0.412	0.416	0.870	0.10
ST	0.414	0.358	0.016^*^	1.17	0.408	0.358	0.029^*^	1.06
BF	0.421	0.364	0.019^*^	1.31	0.421	0.367	0.022^*^	1.29

Table 5 presents the peak amplitude time on EMG for both HAL and control groups. Neither group exhibited statistical significance in the pre- and post-intervention assessments. Nevertheless, it is notable that only the HAL group demonstrated moderate and large effect sizes for VM, VL, and ST.

**Table 5:** Peak amplitude time on EMG for the HAL and control groups The means and corresponding standard deviations are presented. Paired t-tests were employed for the conducted tests, while Wilcoxon's signed-rank test was utilized when applicable (^*^). Effect sizes were computed using Cohen's d. HAL: hybrid assistive limb; post: post-assessment; pre: pre-assessment; VM: vastus medialis; VL: vastus lateralis; BF: biceps femoris; ST: semitendinosus

		HAL group		p-value	Effect size (d)	Control group		p-value	Effect size (d)
		Pre	Post			Pre	Post		
VM	Session 1	0.581±0.25	0.411±0.16	0.096	0.970	0.300±0.22	0.211±0.13	0.317	0.511
	Session 2	0.524±0.47	0.329±0.23	0.305	0.617	0.281±0.22	0.286±0.18	0.959	0.125
	Session 3	0.472±0.12	0.408±0.23	0.517	0.318	0.273±0.14	0.312±0.18	0.792	0.126
VL	Session 1	0.445±0.18	0.473±0.19	0.619	0.241	0.261±0.16	0.157±0.08	0.228	0.636
	Session 2	0.563±0.40	0.375±0.20	0.200	0.686	0.346±0.18	0.377±0.14	0.671	0.205
	Session 3	0.471±0.16	0.345±0.18	0.078	1.056	0.312±0.11	0.162±0.06	0.108	0.924
ST	Session 1	0.167±0.06	0.168±0.08	1.000^*^	0.019	0.319±0.22	0.163±0.05	0.222	0.647
	Session 2	0.208±0.04	0.180±0.03	0.080^*^	0.922	0.160±0.05	0.140±0.02	0.431	0.391
	Session 3	0.191±0.07	0.157±0.03	0.207	0.672	0.159±0.07	0.144±0.02	0.604	0.252
BF	Session 1	0.168±0.06	0.172±0.06	0.893	0.073	0.249±0.18	0.232±0.15	0.686^*^	0.288
	Session 2	0.320±0.29	0.189±0.03	0.345^*^	0.467	0.265±0.16	0.262±0.25	0.500^*^	0.009
	Session 3	0.186±0.06	0.174±0.05	0.582	0.267	0.349±0.28	0.137±0.04	0.165	0.759

In Table 6, model 1 encompassing age, sex, BMI, sports level, injured side, group, pre-post, pre-post, group interaction, and session as fixed effects, was developed for the peak amplitude time of EMG. Additionally, model 2, with fixed effects of age, BMI, and the interaction between group, pre-post, pre-post, and group, and session, was designed and analyzed using GLMM. The results indicated no significant variables for VM in either model 1 or 2. Similarly, no significant variables were identified for the VL. In the case of ST, only the pre-post period demonstrated significance in models 1 and 2. However, for BF, no significant variables were observed in either model 1 or 2. Comparing the information criterion values for AICc and BIC, which were smaller in model 2 for all four peak amplitude times of VM, VL, ST, and BF, the results for model 2 were adopted in all cases. However, no significant variables were identified for the VM, VL, or BF. The pre-post change was significant in the ST. Although no differences were observed between the groups, the p-value for the VM was close to p=0.05, the set significance level.

**Table 6:** GLMM of peak amplitude time on EMG for the HAL and control groups ^*^: significant difference (p<0.05) AICc: Akaike's Information Criterion Corrected; BIC: Schwarz's Bayesian Information Criterion; Group: effects of group (HAL group/control group); pre-post: measurement period; pre-post group: interaction between group and measurement period; VM: vastus medialis; VL: vastus lateralis; BF: biceps femoris; ST: semitendinosus; BMI: body mass index

Variables	VM				VL				ST				BF			
	Model 1		Model 2		Model 1		Model 2		Model 1		Model 2		Model 1		Model 2	
	t-value	p-value	t-value	p-value	t-value	p-value	t-value	p-value	t-value	p-value	t-value	p-value	t-value	p-value	t-value	p-value
Intercept	0.408	0.709	1.018	0.348	0.702	0.533	1.339	0.229	1.425	0.237	2.761	0.008	-0.079	0.942	1.443	0.199
Age	1.704	0.185	0.531	0.615	1.404	0.255	0.528	0.617	1.247	0.297	1.124	0.266	0.421	0.702	-0.075	0.942
Sex	1.642	0.194	-	-	0.882	0.443	-	-	0.146	0.892	-	-	1.536	0.219	-	-
BMI	-2.338	0.101	-1.101	0.313	-2.184	0.117	-1.303	0.240	-1.596	0.209	-1.968	0.054	-0.226	0.836	-0.431	0.682
Sports level	-0.283	0.794	-	-	-0.083	0.939	-	-	1.114	0.337	-	-	0.946	0.411	-	-
Injured side	-1.156	0.329	-	-	-1.196	0.318	-	-	-1.000	0.386	-	-	-0.527	0.634	-	-
Group	2.613	0.053	2.100	0.067	1.884	0.142	1.684	0.131	-0.106	0.919	-0.899	0.373	0.393	0.716	-0.787	0.453
Pre-post	-0.244	0.808	-0.249	0.804	-1.502	0.140	-1.449	0.154	-2.154	0.037^*^	-2.138	0.037^*^	-1.574	0.122	-1.581	0.121
Pre-post group	-1.528	0.134	-1.558	0.126	-0.290	0.773	-0.280	0.781	1.080	0.286	0.983	0.330	0.522	0.604	0.503	0.617
Session 3	-0.172	0.865	-	-	-0.256	0.799	-	-	-1.783	0.081	-	-	-0.088	0.931	-	-
Session 2	-0.301	0.764	-	-	1.894	0.065	-	-	-1.432	0.159	-	-	1.016	0.315	-	-
Model goodness of fit																
AICc	5.856		-4.123		-12.834		-19.886		-66.288		-88.063		-13.371		-26.033	
BIC	9.332		-0.422		-9.311		-16.143		-62.812		-84.363		-9.895		-22.333	

Concerning the interaction, we assessed the contrast between pre and post in each group using the estimated mean (Table 7). Additionally, effect sizes were computed using Cohen's d. The overall estimated marginal means showed significant improvement from pre-treatment to post-treatment. The HAL group exhibited a significant difference in the VM (p=0.019), while VL showed no significant difference but a larger effect size (d=0.61). Conversely, the control group exhibited a significant difference in the ST (p=0.037).

**Table 7:** Overall peak amplitude time on EMG estimated marginal means from pretreatment to posttreatment for the HAL and control groups ^*^: significant difference (p<0.05) HAL: hybrid assistive limb; post: post-assessment; pre: pre-assessment; VM: vastus medialis; VL: vastus lateralis; BF: biceps femoris; ST: semitendinosus; d: effect sizes were computed using Cohen's d

	HAL group		p-value	Effect size (d)	Control group		p-value	Effect size (d)
	Pre	Post			Pre	Post		
VM	0.523	0.375	0.019^*^	0.86	0.292	0.277	0.804	0.20
VL	0.484	0.389	0.071	0.61	0.316	0.241	0.154	0.48
ST	0.188	0.166	0.473	0.46	0.215	0.151	0.037^*^	1.36
BF	0.224	0.182	0.402	0.31	0.292	0.215	0.121	0.58

Table 8 outlines the RER at 0-30, 0-50, and 0-75 ms for both the HAL and control groups. Regarding pre- and post-assessments, neither group demonstrated statistical significance. Notably, the control group reduced their assessments, except for the ST. Conversely, the HAL group exhibited moderate effect sizes. As shown in Table 9, the GLMM analysis conducted for the rate of EMG rise at 0-30, 0-50, and 0-75 ms, considering the variables addressed in model 2 for pre-motor time and peak amplitude time of EMG, revealed that age and BMI were not significant in all analyses. Regarding the group, the interaction between the group and pre-post was significant for RER30, RER50, and RER75 in VM and VL, although it was not significant for the group alone. However, these interactions were not significant in ST and BF.

**Table 8:** RER at 0-30, 0-50, and 0-75 ms for the HAL and control groups The means and corresponding standard deviations are presented. Paired t-tests were employed for the conducted tests, while Wilcoxon's signed-rank test was utilized when applicable (^*^). Effect sizes were computed using Cohen's d. HAL: hybrid assistive limb; post: post-assessment; pre: pre-assessment; VM: vastus medialis; VL: vastus lateralis; BF: biceps femoris; ST: semitendinosus; RER30: rate of EMG rise time intervals of 30 ms; RER50: rate of EMG rise time intervals of 50 ms; RER75: rate of EMG rise time intervals of 75 ms

		HAL group		p-value	Effect size (d)	Control group		p-value	Effect size (d)
		Pre	Post			Pre	Post		
VM	RER30	626.60±1112.9	1322.40±1414.0	0.500^*^	0.339	685.20±448.2	317.00±250.7	0.254	0.595
	RER50	505.16±790.4	1325.20±1252.1	0.345^*^	0.509	703.80±342.5	228.06±162.7	0.053	1.215
	RER75	552.50±517.4	995.60±885.9	0.338	0.487	1008.80±908.3	305.20±297.0	0.043^*^	1.090
VL	RER30	328.28±300.4	635.40±552.9	0.216	0.656	549.60±280.6	359.56±293.2	0.219	0.652
	RER50	473.08±456.5	795.80±820.4	0.351	0.472	790.20±556.3	419.60±222.1	0.108	0.921
	RER75	463.64±414.6	686.60±658.9	0.303	0.528	760.60±408.0	472.60±358.5	0.006	2.398
ST	RER30	196.78±104.4	597.50±572.9	0.179	0.727	232.26±134.0	331.52±294.0	0.587	0.264
	RER50	284.60±153.1	623.94±672.5	0.331	0.494	240.20±140.2	414.80±412.9	0.893^*^	0.347
	RER75	395.20±204.6	750.62±830.2	0.404	0.416	318.60±191.2	442.80±337.4	0.570	0.277
BF	RER30	371.28±292.5	671.56±765.6	0.504	0.328	478.50±274.7	223.82±180.8	0.172	0.743
	RER50	383.40±262.6	916.00±1014.2	0.359	0.463	471.36±308.5	180.30±124.1	0.057	1.187
	RER75	360.20±246.1	888.60±1012.4	0.279^*^	0.466	421.06±281.1	275.62±246.4	0.127	0.858

**Table 9:** GLMM of the rate of EMG rise for the HAL and control groups ^*^: significant difference (p<0.05) AICc: Akaike's Information Criterion Corrected; BIC: Schwarz's Bayesian Information Criterion; group: effects of group (HAL group/control group); pre-post: measurement period; pre-post group: interaction between group and measurement period; VM: vastus medialis; VL: vastus lateralis; BF: biceps femoris; ST: semitendinosus; BMI: body mass index; RER30: rate of EMG rise time intervals of 30 ms; RER50: rate of EMG rise time intervals of 50 ms; RER75: rate of EMG rise time intervals of 75 ms; EMG: electromyography; HAL: hybrid assistive limb

Variables	VM						VL					
	RER30		RER50		RER75		RER30		RER50		RER75	
	t-value	p-value	t-value	p-value	t-value	p-value	t-value	p-value	t-value	p-value	t-value	p-value
Intercept	-0.084	0.934	-0.022	0.983	-0.004	0.997	-0.008	0.994	0.000	1.000	-0.003	0.997
Age	0.077	0.940	0.022	0.983	0.009	0.993	0.016	0.988	0.001	1.000	0.004	0.997
BMI	-0.003	0.998	-0.001	0.999	-0.001	0.999	-0.002	0.998	0.009	0.993	0.009	0.993
Group	-0.026	0.979	-0.012	0.991	-0.010	0.992	-0.015	0.988	-0.009	0.993	-0.011	0.991
Pre-Post	-1.530	0.165	-1.983	0.083	-2.133	0.065	-1.636	0.140	-1.385	0.204	-1.858	0.100
Pre-Post Group	2.492	0.037^*^	2.961	0.018^*^	2.863	0.021^*^	2.593	0.032^*^	2.331	0.048^*^	2.647	0.029^*^
Model goodness of fit												
AICc	209.601		287.672		285.794		264.721		275.517		269.331	
BIC	279.788		287.860		285.982		264.908		275.704		269.518	

Variables	ST						BF					
	RER30		RER50		RER75		RER30		RER50		RER75	
	t-value	p-value	t-value	p-value	t-value	p-value	t-value	p-value	t-value	p-value	t-value	p-value
Intercept	-0.030	0.977	-0.030	0.976	-0.025	0.981	-0.018	0.986	-0.024	0.981	-0.017	0.987
Age	0.025	0.980	0.024	0.981	0.019	0.985	0.008	0.994	0.015	0.988	0.010	0.992
BMI	0.002	0.999	0.001	0.999	0.003	0.998	0.019	0.985	0.012	0.991	0.009	0.993
Group	0.013	0.989	0.021	0.984	0.017	0.986	0.007	0.994	0.015	0.988	0.010	0.992
Pre-Post	-0.386	0.710	0.494	0.634	0.029	0.978	-1.947	0.087	-1.795	0.110	-1.237	0.251
Pre-Post Group	0.456	0.661	-0.866	0.411	-0.475	0.647	1.479	0.177	1.697	0.128	1.393	0.201
Model goodness of fit												
AICc	260.484		267.145		272.637		268.139		270.632		274.116	
BIC	260.672		267.332		272.824		268.326		270.820		274.303	

Concerning the interaction, we assessed the contrast between pre and post in each group using the estimated mean (Table 10). Additionally, effect sizes were computed using Cohen's d. The overall estimated marginal means showed significant improvement from pre-treatment to post-treatment, revealing a significant difference in ST-RER30 in the HAL group (p=0.044). Additionally, although no significant differences were identified, there were five instances where the effect size exceeded 0.8. Conversely, in the control group, no significant difference was observed, and the effect size exceeded 0.80 only in VM-RER75.

**Table 10:** Overall rate of EMG rise estimated marginal means from pretreatment to posttreatment for the HAL and control groups ^*^: significant difference (p<0.05) HAL: hybrid assistive limb; post: post-assessment; pre: pre-assessment; VM: vastus medialis; VL: vastus lateralis; BF: biceps femoris; ST: semitendinosus; RER30: rate of EMG rise time intervals of 30 ms; RER50: rate of EMG rise time intervals of 50 ms; RER75: rate of EMG rise time intervals of 75 ms; d: effect sizes were computed using Cohen's d

		HAL group		p-value	Effect size (d)	Control group		p-value	Effect size (d)
		Pre	Post			Pre	Post		
VM	RER30	663.32	1359.12	0.268	0.73	648.48	280.28	0.551	0.39
	RER 50	535.47	1355.51	0.118	1.05	673.49	197.75	0.351	0.61
	RER 75	570.09	1013.19	0.245	0.59	991.22	282.62	0.081	0.93
VL	RER 30	340.24	647.36	0.116	0.77	537.64	347.60	0.308	0.48
	RER 50	490.86	813.58	0.234	0.53	772.42	401.82	0.178	0.61
	RER 75	485.73	708.69	0.147	0.44	738.51	450.51	0.072	0.57
ST	RER 30	218.36	619.08	0.044^*^	1.40	210.68	309.94	0.592	0.11
	RER 50	308.98	648.32	0.142	0.98	215.82	390.42	0.437	0.51
	RER 75	426.45	781.87	0.177	0.90	287.35	411.55	0.627	0.31
BF	RER 30	400.52	700.80	0.264	0.74	449.26	194.58	0.340	0.62
	RER 50	420.00	952.60	0.107	1.09	434.77	143.71	0.363	0.59
	RER 75	397.78	926.18	0.110	1.08	383.48	238.04	0.646	0.30

## Discussion

Regarding the interaction of pre-motor time within each group using the estimated mean, significant differences were noted in ST and BF in both groups, with no significant differences in VM. Notably, the effect size of VM was larger in the HAL group. For the peak amplitude time of EMG, it is notable that only the HAL group demonstrated moderate and large effect sizes for VM, VL, and ST. The analysis confirmed the contrast between the pre- and post-intervention for each group based on the estimated mean. The HAL group exhibited a significant difference in the VM, while the VL showed no significant difference but had a larger effect size. In terms of RER, regarding the group, the interaction between the group and pre-post was significant for all RER30, RER50, and RER75 in the VM and VL, although it was not significant for the group alone. A comparison between the pre- and post-tests for each group based on the estimated mean revealed a significant difference in the ST-RER30 in the HAL group. Additionally, although no significant differences were identified, there were five instances where the effect size exceeded 0.8. This study utilized EMG to analyze the temporal component, focusing on the reaction time of muscle activity and the subsequent time to maximal muscle activity. In a previous study involving knee HAL single-joint training for patients after ACL reconstruction, notable differences in muscle strength were observed compared to the control group, particularly across various velocities in isokinetic muscle strength tests [19]. Optimization of muscle function has been attributed to potential improvements in neuromuscular coordination, reduction in muscle stiffness, and addressing deficits in the spinal reflex pathways [2,30,31]. However, previous research has primarily focused on physical assessments and isokinetic muscle strength and lacks electrophysiological examinations. Therefore, the findings of the current study emphasize the importance of incorporating neurophysiological perspectives to obtain a more comprehensive understanding of the effects and underlying mechanisms involved, despite the available evidence suggesting that knee HAL single-joint training can positively affect muscle strength recovery. Prolonged reaction times may indicate potential influences of concurrent proprioceptive disturbances involving processing time intervals in the central nervous system [2,24,32]. This delay may result from defects in neural processing during force generation, physiological peripheral muscle changes, or a combination of both [2,24]. Although the small number of participants prevented the identification of statistically significant differences, moderate-to-large effect sizes were observed in the HAL group, suggesting an immediate transformative effect. The physiological mechanisms implicated in the measurements include muscle reaction time, which reflects central nervous system processing (nerve conduction and transmission through the spinal cord, peripheral nerve transmission, and muscle action potential transmission) [2]. Similarly, peak force time and peak force duration may reflect factors such as the contraction of motor units and bone transmission of muscle torque [33]. Maximal voluntary rates of neuromuscular activation have been reported at the onset of rapid muscle contractions, as evidenced by the slope of the EMG signal, motor unit firing rates, and incidence of doublet firing [34-36]. Given that HAL training involves both voluntary automatic and joint torque movements, mutual feedback can occur between the muscles, joints, ligaments, and other tissues during training. This interaction may contribute to the neurophysiological changes and musculoskeletal effects of joint movement training, as suggested by the results of this study. A previous study suggested that feedback from potential users is essential during the development of technologies for rehabilitation medicine [37]. HAL utilizes a technology that combines voluntary drive and normalized motion assistance from an external device. This is based on the user's motion intention and the appropriate sensory input evoked by HAL-supported motion [15]. This is particularly beneficial for patients with spinal reflex pathway lesions involving reciprocal inhibition or γ-motor and α-motor neurons. Neural activity combined with repetitive task execution promotes and potentially restores or restructures appropriate proprioceptive feedback learning [38]. Another study employed integrative electrophysiological and molecular approaches to comprehensively explore alterations in neuromuscular integrity and function after 10-day unilateral lower limb suspension, followed by 21 days of active recovery in young healthy men. Intramuscular EMG analysis indicated increased motor-unit potential complexity and decreased motor-unit firing rates following unloading, which are potentially associated with changes in the skeletal muscle ion channel pool and initial signs of fiber denervation and axonal damage [39].

This study has some limitations. First, examining its reliability in different populations is essential to ascertain the clinical application of the total reaction time, pre-motor time, and motor time. Additionally, the sample size in this study was limited to five participants in each group despite the significant differences revealed by the GLMM post-hoc test. A comprehensive study design should be developed by determining the required sample size based on the findings of this study. The trial's sample size is relatively small; thus, it is crucial to acknowledge the potential impact of the limited number of participants on the overall robustness of the study. While sample-size design based on hypothesis testing is common in many clinical studies, exploratory studies may also use sample-size design in terms of avoiding administering unvalidated treatments to large populations or considering feasibility [40]. One way to address this problem is the Bayesian decision theory. In this study, the potential for analysis was explored using GLMM, a hierarchical Bayesian modeling approach. Consequently, a GLMM post hoc test was conducted. Regarding the interaction of pre-motor time within each group using the estimated mean, significant differences were noted in ST and BF in both groups, with no significant differences in VM. Notably, the effect size for VM was larger in the HAL group.

## Conclusions

The EMG results indicate that knee HAL training for post-ACL reconstruction patients may influence neurophysiological outcomes. Specifically, it appears to alter the reaction time and duration of muscle activity. This study suggests that HAL training has positive effects on the neuromuscular aspects of patients recovering from ACL reconstruction.
